# Development of anti-PD-L1 antibody based on structure prediction of AlphaFold2

**DOI:** 10.3389/fimmu.2023.1275999

**Published:** 2023-10-24

**Authors:** Kun Du, He Huang

**Affiliations:** ^1^ School of Chemical Engineering and Technology, Tianjin University, Tianjin, China; ^2^ Frontiers Science Center for Synthetic Biology and Key Laboratory of Systems Bioengineering (Ministry of Education), Tianjin University, Tianjin, China

**Keywords:** AlphaFold2, artificial intelligence, antibody, programmed death-ligand 1, programmed cell death protein 1, cancer immunotherapy

## Abstract

Accurate structural information plays a crucial role in comprehending biological processes and designing drugs. Indeed, the remarkable precision of the AlphaFold2 has facilitated significant advancements in predicting molecular structures, encompassing antibodies and antigens. This breakthrough has paved the way for rational drug design, ushering in new possibilities in the field of pharmaceutical development. Within this study, performing analysis and humanization guided by the structures predicted by AlphaFold2. Notably, the resulting humanized antibody, h3D5-hIgG1, demonstrated exceptional binding affinity to the PD-L1 protein. The KD value of parental antibody 3D5-hIgG1 was increased by nearly 7 times after humanization. Both h3D5-hIgG1 and 3D5-hIgG1 bound to cells expressing human PD-L1 with EC50 values of 5.13 and 9.92nM, respectively. Humanization resulted in a twofold increase in the binding capacity of the antibody, with h3D5-hIgG1 exhibiting superior performance compared to the parental antibody 3D5-hIgG1. Furthermore, h3D5-hIgG1 promoted cytokine secretion of T cells, and significantly suppressed MC38-hPD-L1 tumor growth. This study highlights the potential for artificial intelligence-assisted drug development, which is poised to become a prominent trend in the future.

## Introduction

On July 16, 2021, the DeepMind team published a study titled “Highly accurate protein structure prediction with AlphaFold” in the scientific journal Nature, disclosing the source code of AlphaFold2 in CASP14 (1). In the CASP14 experiment, AlphaFold2 demonstrated significantly higher accuracy in protein structure prediction compared to competing methods. On July 22, the team published “Highly accurate protein structure prediction for the human proteome”, which described the successful prediction of human proteome structure by AlphaFold2 with high reliability, covering approximately 58% of amino acids in the human proteome (2).

Structure prediction has seen substantial progress in recent years, as evidenced by the results of the biennial Critical Assessment of protein Structure Prediction (CASP) (3, 4). Accurate structural information is crucial for understanding biological processes and drug design. Until now, high-resolution crystal structures have been the primary basis for protein structure-based drug discovery. However, experimentally identified structures cover only approximately 17% of the amino acids in the human proteome. The AlphaFold2 system has demonstrated the ability to predict the structural positions of approximately 58% of the amino acids in the human proteome with high reliability. Of these, 35.7% were predicted with high confidence, which is twice the number of structures covered by the experimental method. At the protein level, AlphaFold2 predicted at least three-quarters of the amino acid sequences of 43.8% of the proteins. This accurate structure prediction by AlphaFold2 has significantly expanded the accessibility of rational drug design.

Protein structure prediction enables the provision of actionable structural hypotheses rapidly and on a large scale, which helps to address the gap in structural knowledge. Previous large-scale structure prediction studies have addressed protein families (5–8), specific functional classes (9, 10), domains within whole proteomes (11), and in some cases, full chains or complexes (12, 13).

Immune checkpoints are inhibitory pathways of the immune system that maintain self-tolerance and prevent autoimmunity (14–18). However, tumor cells often upregulate these immune checkpoints to induce local immune suppression and attenuate the endogenous antitumor immune response (19, 20). PD-L1, for example, is often overexpressed in various tumors, such as melanoma, lung, and breast cancer, leading to immune response inhibition in the tumor microenvironment (20, 21). The PD-1/PD-L1 interaction inhibits T-lymphocyte proliferation, cytokine release, and cytotoxicity, causing exhaustion and apoptosis of tumor-specific T cells. However, blocking the PD-1/PD-L1 interaction results in the reversal of the exhausted T-cell phenotype and normalization of the antitumor response (22, 23).

**Figure 1:**
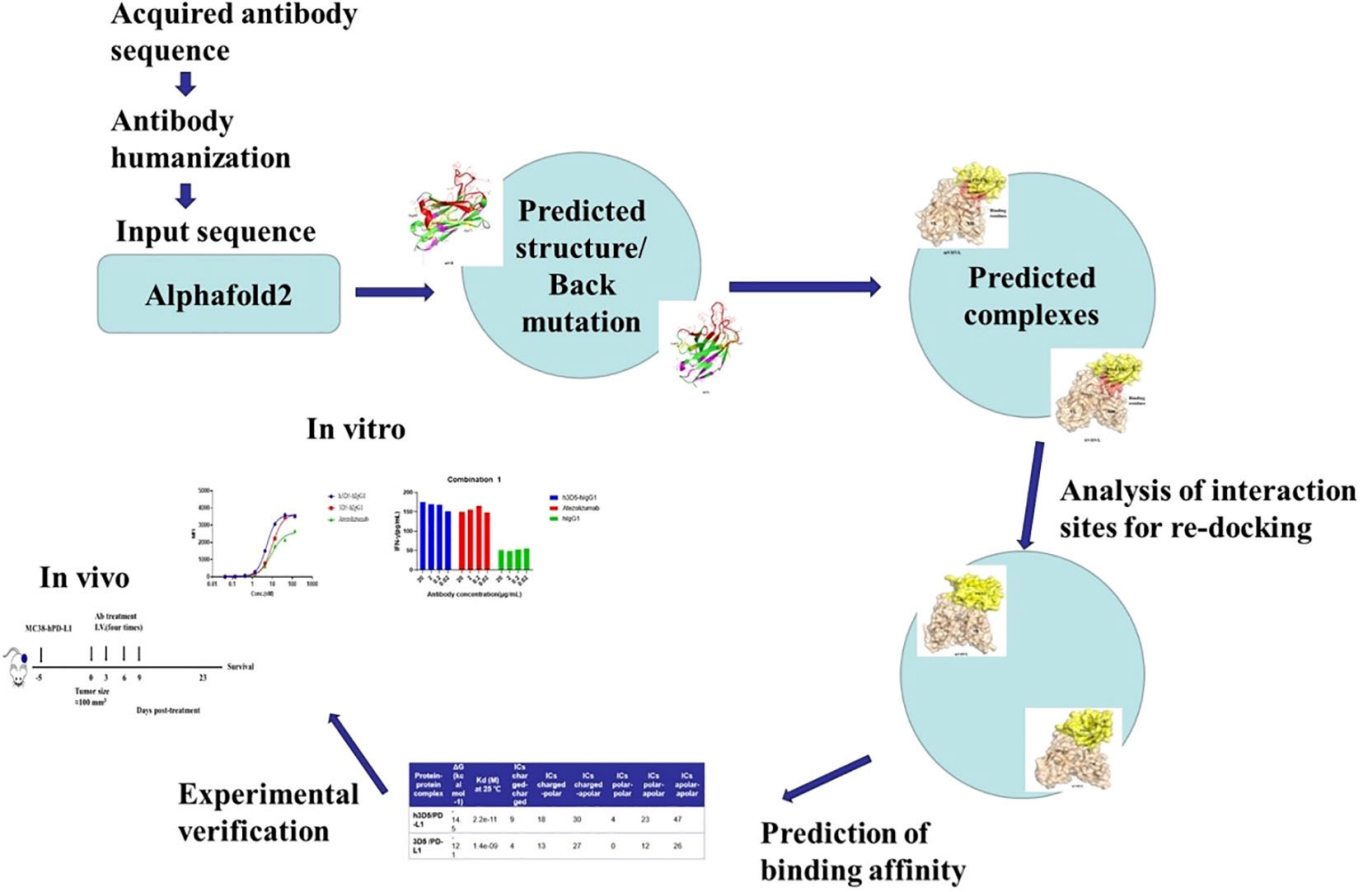
Framework diagram.

In this study, the antibody and antigen structures were predicted with AlphaFold2, and the antibody was promptly and accurately humanized, resulting in the successful production of an excellent anti-PD-L1 antibody, h3D5-hIgG1. To facilitate comprehension, a research framework diagram has been formulated, seamlessly integrating computational and experimental aspects, as illustrated in **Figure 1**. Our findings suggest that h3D5-hIgG1 is a promising therapeutic candidate for the treatment of cancer. It demonstrated excellent inhibitory effects on tumor growth in syngeneic tumor models.

## Results

### Screening and identification of anti-human PD-L1 antibody

BALB/C mice were immunized multiple times with the extracellular region of PD-L1 until the optimal serum titer was achieved. Through the use of hybridoma technology, the 3D5-mIgG anti-PD-L1 antibody was generated. To determine its binding activity by 293T-PD-L1 cells, 3D5-mIgG was assessed for its ability to block the binding of PD-1 and PD-L1. 3D5-mIgG demonstrated effective blocking of this interaction. The binding capacity of the 3D5 antibody on 293T-PD-L1 cells was found to be greater than that of atezolizumab. However, the 3D5 antibody showed slightly weaker blocking ability compared to atezolizumab. After sequencing, the Fc fragment of human IgG1 was fused to the 3D5-mIgG antibody to generate the chimeric 3D5-hIgG1 antibody, which exhibited favorable characteristics. Further humanization was performed on the 3D5-hIgG1 antibody.

### Design of humanized VH/VL and structure modeling

The variable regions of an antibody’s heavy and light chains (VH and VL) form domains that include three complementarity-determining regions (CDRs 1-3) and four framework regions (FRs 1-4) belonging to the immunoglobulin superfamily. The concept of generating less immunogenic antibodies through CDR grafting originated from the hypothesis that replacing the CDRs of a human antibody (the acceptor) with those of a mouse monoclonal antibody (the donor) would not affect the antigen binding site formed by the mouse CDRs.

Align each parental nonhuman antibody framework sequence(heavy chain framework 1, 2, 3, and 4 and light chain framework 1, 2, 3, and 4) with the human germline framework sequences obtained through the protein database, such as NCBI. Taking into consideration the antibody’s sequence similarity with various human germline templates, expression levels, and whether the combination of heavy and light chains has been utilized by existing therapeutic antibodies, a comprehensive assessment was performed. Ultimately, The human germline gene templates were chosen as templates for the heavy and light chains: IGHV1-46*01 and IGKV1-5*01, respectively.

**Figure 2:**
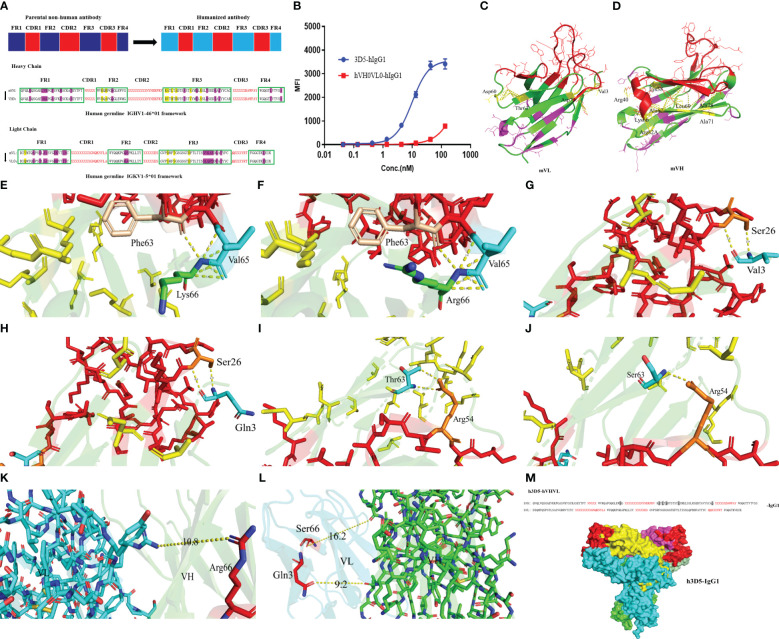
Structure prediction. **(A)** Selecting the acceptor human framework by framework shuffling. **(B)** Binding of hVH0VL0-hIgG1 to cell surface human PD-L1 was determined by FACS. **(C)** mVH structure **(D)** mVL structure **(E)** Lys66 in the HFR3 interacts with Val65 and Phe63 in the HCDR2 of VH structure of the parental nonhuman antibody 3D5 **(F)** Mutating Lys66 in the parental nonhuman antibody 3D5 to Arg67 **(G)** The interaction between Val3 and Ser26 in the LCDR1 of VL structure of the Parental Nonhuman Antibody 3D5 **(H)** Mutating Val3 from Parental Nonhuman Antibody 3D5 to Gln3 **(I)** The interaction between Thr66 and Arg54 in the LCDR1 of VL structure of the Parental Nonhuman Antibody 3D5 **(J)** Mutating Thr66 from Parental Nonhuman Antibody 3D5 to Ser66 **(K, L)** The distance between amino acid residues that were modified in the VH/VL **(M)** The humanized antibody VHVL sequence and the full-length structure.

Grafting of mouse antibody CDRs into the human germline framework, as illustrated in the provided **Figure 2A**. The preliminary humanized antibody sequences resulting from this process were designated as hVH0VL0. Synthesized cDNA fragments encompassing the full-length heavy and light chain regions, including the signal sequence, humanized variable region sequence, and human constant region sequence, were introduced into a mammalian cell expression vector. This construction facilitated the development of designed expression vectors for the humanized antibody heavy chain and light chain, respectively. The accuracy of the nucleotide sequences within the prepared expression vectors was validated through DNA sequencing. Subsequently, the humanized antibody was transiently expressed using the FreeStyle 293 Expression System. Upon Validation of the activity of the humanized antibody, a decrease in activity was observed, as depicted in **Figure 2B**. It is hypothesized that mutations in specific amino acid residues during the CDRs grafting process may have impaired the functionality of the antibody’s complementary determining regions.

Subsequently, we employed AlphaFold2 to perform structural predictions on the variable regions of both the heavy and light chains of the antibody. We conducted a thorough analysis of the resultant structures and proceeded with revertant mutagenesis experiments. Using PyMOL, we analyzed the structures of VH and VL. Notably, the residues comprising the upper hydrophobic core within the immunoglobulin domain were found to be involved in interactions with amino acids in the CDRs, as a platform of residues directly underneath the CDRs. This interaction provides support for the conformation of the amino acids within the CDRs. Specifically, the upper hydrophobic core residues are located at the following positions: 2, 4, 24, 27, and 29 in framework 1; 47-49 in framework 2; 69, 71, 78, and 94 in framework 3 of the heavy chain; and positions 2 and 4 in framework 1; and 64, 66, and 71 in framework 3 of the light chain of the original nonhuman antibody framework sequence (residue positions are described in Kabat numbering). For each parental nonhuman antibody framework, we selected a human germline framework sequence that entirely preserves all upper hydrophobic core residues. In cases where such human germline framework sequences meeting these criteria are not available, it is necessary to perform back mutations on these upper hydrophobic core residues. This step is crucial to prevent any potential impact on the conformation and functionality of the CDRs.

Examining interactions within 4.2Å, as depicted in **Figure 2C**, within the heavy chain VH, interactions occur between Lys38, Arg40, Vel48, Lys66, Ala67, Leu69, Ala71, Ala78, Arg82A, and Gly94 residues with amino acid residues in the CDRs, highlighted in yellow. The CDR region is indicated in red. Purple amino acid residues do not engage in interactions with amino acid residues in the CDRs. Mutations to these residues, which are typically conserved germline framework residues, are not anticipated to affect antibody-antigen interactions. Generally, amino acid residues that are exposed on the surface of the antibody and are distant from the CDRs do not affect the antigen-binding activity. As such, back mutation is unnecessary for these residues. Of the upper hydrophobic core residues, namely Vel48, Ala67, Leu69, Ala71, Ala78, and Gly94, they are retained and subjected to back mutation during the humanization process. It is essential to analyze whether mutations to Lys38, Arg40, Kys66, and Arg82A would affect the functionality of the CDRs.

Among the targeted amino acid residues for mutation, it was observed that Lys66 in the heavy chain framework region 3 (HFR3) interacts with Val65 and Phe63 in the heavy chain complementarity-determining region 2 (HCDR2), as shown in **Figure 2E**. In the human germline framework, the 66th amino acid residue is Arg. Because Lys and Arg have similar properties as positively charged basic amino acids, Arg66 could still interact with Val65 and Phe63 after mutation, as shown in **Figure 2F**, without other effects on the CDR region. Reversion mutagenesis is not necessary for Arg66. The mutations of Lys38 to Arg38, Arg40 to Ala40, and Arg82A to Ser82A result in relatively minor effects on the CDRs. Therefore, considering the weak impact, back mutations may not be necessary.

For the light chain VL, select the human germline gene IGKV1-5*01 framework sequence that fully retains all the upper hydrophobic core residues. Notably, interactions were observed between Val3, Asp60, Thr63, and Asp70 residues and amino acid residues within the Complementary Determining Regions (CDRs), as illustrated in **Figure 2D**.

In the human germline framework sequence, position 3 is Gln, and position 63 is Ser, while other amino acid residues did not affect CDR and were modified normally. As shown in **Figures 2G, H**, the interaction between Val3, Gln3, and Ser26 in CDR1 of VL is similar in the three-dimensional structure. However, the interaction force formed by Ser63 and Arg54 after modification was one hydrogen bond less than that formed by Thr63 and Arg54 in parental nonhuman antibody framework, as shown in **Figures 2I, J**. That is because serine lacks a methyl group compared to threonine. Considering the similarity in properties between serine and threonine, Thr66 was still mutated to Ser66. The substitutions of Asp60 to Ser60 and Asp70 to Glu have no significant impact on the CDRs. Therefore, back mutations are not required. This implies that no modifications may be required for the light chain germline framework.

Through VH/VL three-dimensional structure prediction, as shown in **Figures 2K, L**, it was observed that the modification of amino acid residues did not affect the interaction between VH and VL. For hydrogen bonds, the distance between donor and acceptor atoms is usually 2.7-3.3Å. Hydrophobic interactions (van der Waals bonds) have carbon-carbon distances a bit longer, usually 3.3-4.0Å. The distance between amino acid residues was longer than 10Å, and no interaction force could be formed. The humanized antibody VHVL sequence is illustrated in **Figure 2M**, with the full-length structure predicted by AlphaFold2. The gray background signifies the amino acid residues that underwent back mutations. Finally, the humanized antibody, h3D5-hVHVL-hIgG1, abbreviated as h3D5-hIgG1, was determined.

### Predicting the structural configuration of 3D5 (h3D5)/PD-L1 complexes

**Figure 3:**
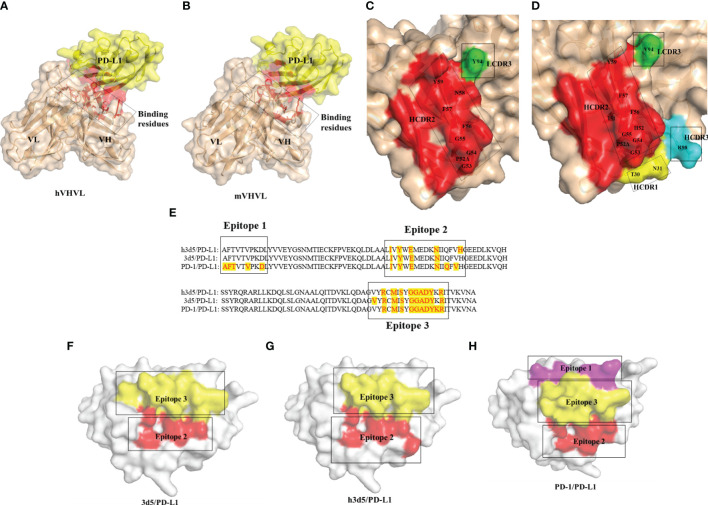
Overall structure of the 3D5(h3D5)/PD-L1 complexes **(A)** 3D5/PD-L1 complex The red labels indicate the regions of interaction in the 3D5/PD-L1 complex. **(B)** h3D5/PD-L1 complex The red labels indicate the regions of interaction in the h3D5/PD-L1 complex. **(C)** Open view of the binding surfaces of 3D5 The red labels represent the amino acids in the HCDR2 region of the mVHVL(The VHVL of the parental murine antibody). The green labels represent the amino acids in the LCDR3 region of the mVHVL. **(D)** Open view of the binding surfaces of h3D5 The red labels represent the amino acids in the HCDR2 region of the hVHVL(The VHVL of the humanized antibody). The yellow labels represent the amino acids in the HCDR1 region of the hVHVL. The light blue labels represent the amino acids in the HCDR3 region of the hVHVL. The green labels represent the amino acids in the LCDR3 region of the hVHVL. **(E)** PD-L1 residues that bind h3D5, 3D5 and PD-1 are highlighted with red tag **(F–H)** Open view of the binding surfaces of PD-L1 Corresponding to e.

To investigate whether the humanized antibody affects antigen binding, we predicted the 3D5/PD-L1 complex using AlphaFold2. Alterations to the antibody framework can often result in changes to its binding ability, so we examined whether the interaction between the antibody and antigen was impacted by modification. As shown in **Figures 3A, B**, both the humanized and parental mouse antibodies bound to PD-L1 primarily through the VH region, with the amino acid residues of the VH CDR2 providing the main interaction force, viewed through pymol. Additionally, the red-labeled amino acid residues in **Figures 3C, D** indicated that h3D5 had more binding amino acid residues involved in forming interaction forces with the PD-L1 antigen compared to parental mouse antibody 3D5, suggesting that h3D5 may have superior binding ability. Furthermore, h3D5 exhibited additional interaction forces with PD-L1 epitopes, including HCDR1 Thr30, Asn31, and HCDR3 Arg98, expanding the area of the HCDR2 binding epitopes.

Both 3D5 and h3D5 primarily bound to Epitope 2 and Epitope 3 of the PD-L1 protein, which contained three epitopes that bound to the PD-1 receptor, as shown in **Figure 3E**. The binding epitope residues on the PD-L1 protein were only slightly altered after modification, as seen in **Figures 3F–H**. These findings suggest that the modified antibodies may possess improved binding and blocking abilities due to their enhanced binding affinity.

### Antibody-antigen docking and binding affinity prediction

However, during the process of utilizing AlphaFold2 to predict other antigen-antibody complexes, it should be noted that successful predictions are not attainable for all such complexes. As commonly recognized, the self-learning capabilities of artificial intelligence heavily depend on the availability of a sufficiently robust database. Regarding the prediction of PD-L1 antibody-antigen complexes, the existence of specific antibody-antigen complex crystal structures within the PDB database may have served as valuable resources for AlphaFold2’s learning, potentially enabling successful predictions for this particular class of antibody-antigen complexes. In light of these circumstances, we tried to undertake a new round of Antibody-antigen Docking simulations, according to shape complementarity, electrostatics.

**Figure 4:**
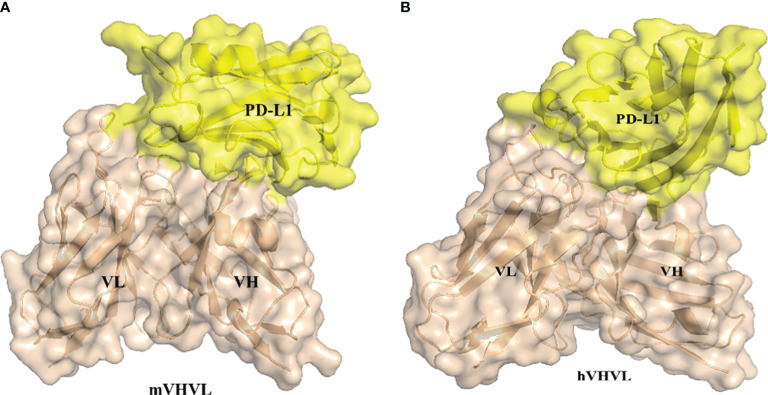
Antibody-antigen Docking using ZDOCK **(A)** 3D5/PD-L1 complex **(B)** h3D5/PD-L1 complex.

The docking of antibody antigens was carried out using ZDOCK (24, 25). Selected binding site residues, referred to alphafold2 for predicting the results of the interactions involving amino acid residues. ZDOCK is a rigid-body protein-protein docking program that provides a user-friendly web interface for producing models of protein-protein complexes and symmetric multimers in a fast and effective manner. In addition to generating and viewing structures of docking models through the server’s tools and interface, users could submit ZDOCK Server output files directly to several available docking refinement and post-processing tools (linked from the server page). The output PDB file is shown in **Figure 4**.

We used PRODIGY (26, 27) to predict the affinity of the antibodies. PRODIGY (PROtein binDIng enerGY prediction) is a collection of web services focused on the prediction of binding affinity in biological complexes as well as the identification of biological interfaces from crystallographic one. The structural properties contributing to binding affinity (BA) have traditionally been assessed by many methods, which primarily focused on the impact of buried surface area (BSA) while overlooking the substantial influence of non-interacting surface (NIS) on binding affinity. The developers of PRODIGY further categorized these properties based on the amino acid type, distinguishing between polar and apolar amino acids, for both BSA and NIS. Additionally, they considered various contact types for inter-residue contacts (ICs), including polar/polar, polar/charged, polar/apolar, charged/charged, charged/apolar, and apolar/apolar interactions. They used the protein–protein BA benchmark consisting of 144 non-redundant protein–protein complexes with experimentally determined Kd (ΔG) and available 3D structures. To use any of the PRODIGY tools, just need to provide the 3D structure of the complex/complexes in PDB/mmCIF format or the ID of its PDB entry. Viewed results directly on the web page. At room temperature, h3D5 has a stronger affinity, with a Kd of 2.2×10^-11^M, while 3D5 has a Kd of 1.4×10^-9^M. The predicted value of the binding free energy (ΔG) is -14.5kcal/mol for h3D5 and -12.1kcal/mol for 3D5. The h3D5/PD-L1 complex generated more intermolecular interactions in terms of the number and type of contacts within the 5.5 Å distance cutoff. Based on the predicted data, the humanized h3D5 antibody has better binding ability, as showed in **Table 1**.

**Table 1:** Binding affinity and Kd prediction and prediction details.

Protein-protein complex	ΔG (kcal mol-1)	Kd (M) at 25 °C	ICs charged-charged	ICs charged-polar	ICs charged-apolar	ICs polar-polar	ICs polar-apolar	ICs apolar-apolar
h3D5/PD-L1	-14.5	2.2×10^-11^	9	18	30	4	23	47
3D5/PD-L1	-12.1	1.4×10^-9^	4	13	27	0	12	26

ICs, Number of Interfacial Contacts.

### Validation and characterization of humanization anti-PD-L1 antibody

**Figure 5:**
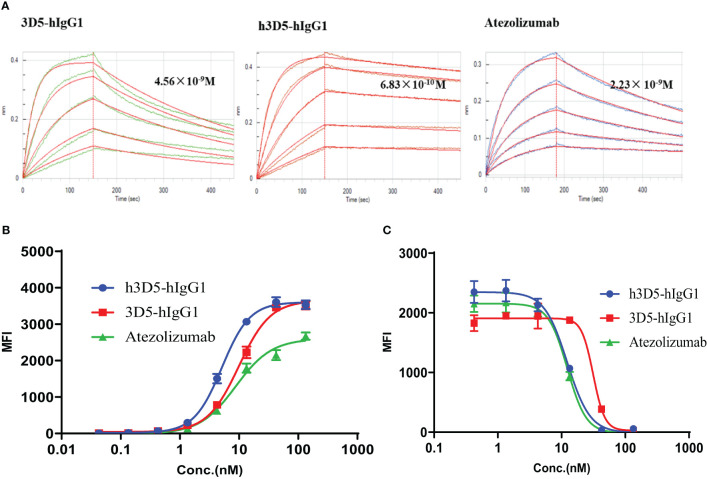
Anti-PD-L1 antibodies bind to the PD-L1 protein and inhibit the interactions between PD-1 and PD-L1. **(A)** BLI assay **(B)** Binding of h3D5-hIgG1 or Atezolizumab to cell surface human PD-L1 was determined by FACS. **(C)** The functionality of h3D5-hIgG1 or Atezolizumab in blocking human PD-1/PD-L1 interactions was assessed by flow cytometry.

The humanized antibody, h3D5-hIgG1, was characterized and found to have potent binding to human PD-L1, with a KD value of 6.83×10^-10^ M, as compared to 3D5-hIgG1 and atezolizumab with KD values of 4.56×10^-9^M and 2.23× 10^-9^M, respectively (**Table 2**), as shown in **Figure 5A**. Using AlphaFold2, it was observed that the KD value of 3D5-hIgG1 increased by nearly 7 times after humanization. H3D5-hIgG1, 3D5-hIgG1, and atezolizumab were also found to bind to cells expressing human PD-L1 with EC50 values of 5.13, 9.92, and 8.8nM, respectively. The humanized antibody h3D5-hIgG1 demonstrated a two-fold increase in binding capacity compared to the chimeric antibody 3D5-hIgG1, which was attributed to more binding amino acid residues in h3D5 that interacted with the PD-L1 antigen.

**Table 2:** Affinity of anti-PD-L1 antibodys to hPD-L1 measured by BLI(ForteBio Octet).

Antibody	Antigen	kdis(1/s)	KD (M)
Atezolizumab	hPD-L1	7.96×10^-4^	2.23×10^-9^
3D5-hIgG1	hPD-L1	1.74×10^-3^	4.56×10^-9^
h3D5-hIgG1	hPD-L1	3.58×10^-4^	6.83×10^-10^

Further, in a competitive flow cytometry assay, h3D5-hIgG1, 3D5-hIgG1, and a reference antibody were found to effectively block the interaction between PD-L1 and PD-1, with IC50 values of 12.3, 31.4, and 12.3nM, respectively. These results demonstrate that the modified h3D5-hIgG1, with the assistance of AlphaFold2, exhibited superior binding and blocking ability, as depicted in **Figures 5B, C**.

### H3D5-hIgG1 enhanced cytokine secretion of T cells

**Figure 6:**
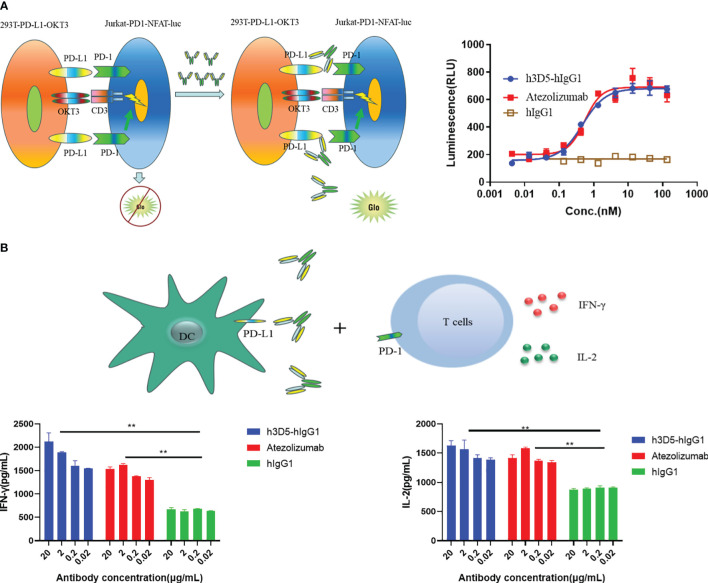
Reporter assay and MLR assay. **(A)** Reporter assays **(B)** The functionality of h3D5-hIgG1 and atezolizumab in enhancing T cell responses was assessed using MLR assay, and effector function (IFN-γ, IL-2 production) were quantified (**p<0.01).

In the report cell line analysis experiment utilizing a two-cell co-culture system comprising a signal sensor cell (Jurkat) expressing the chimeric PD-1 receptor and NFAT-luciferase, and a signal sending cell (293T/PD-L1/OKT3), engagement of PD-1 with PD-L1 led to inhibition of luciferase expression, which was subsequently reversed upon addition of anti-PD-L1 antibodies. In this system, h3D5-hIgG1 and reference ab demonstrated comparable ability to reverse the luciferase expression, as shown in **Figure 6A**.

Blocking the PD-L1 interaction with its receptor resulted in enhanced cytokine secretion of T cells (28). Hence, the capacity of h3D5-hIgG1 to modulate T cell function was assessed through the MLR assay. H3D5-hIgG1 significantly increased IL-2 and IFN-γ levels in T cells in a dose-dependent manner similar to the reference ab, as depicted in **Figure 6B**. In summary, h3D5-hIgG1 promotes cytokine secretion of T cells *in vitro*.

### H3D5-hIgG1 significantly inhibited MC38-hPD-L1 tumor growth

**Figure 7:**
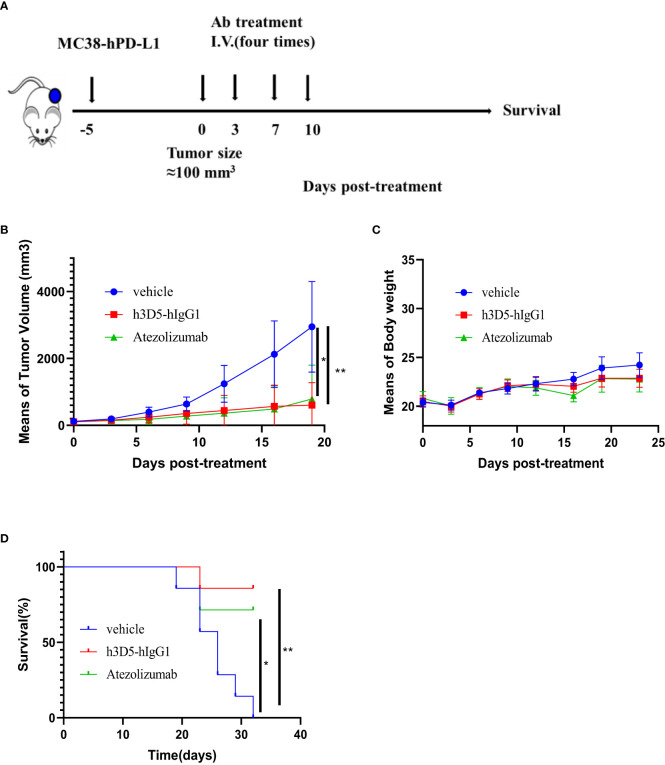
The *in vivo* efficacy of h3D5-hIgG1 was evaluated in an established MC38-hPD-L1 model. **(A)** Humanized transgenic mice expressing human PD-L1 (n=7 per group) were subcutaneously inoculated with MC38-hPD-L1 tumor cells. Five days after the injection of tumor cells, when the average tumor size reached approximately 100 mm^3^, mice with established tumors were randomly divided into various treatment groups. These groups received specific antibodies at a dose of 10 mg/kg, administered four times. **(B)** Anti-human PD-L1 antibody inhibited MC38-PD-L1 tumor growth. Asterisks indicate statistical significance (*p=0.0142, **p=0.0025) **(C)** Body weight changes during treatment **(D)** Survival curve of the mice in the groups. Asterisks indicate statistical significance (*p=0.0202, **p=0.002).

The therapeutic effectiveness of h3D5-hIgG1 was assessed in the MC38-hPD-L1 colon cancer model using hPD-L1 knock-in mice(**Figure 7A**). Mice were divided into groups when the tumor volume reached approximately 100 mm^3^, and subsequently treated with anti-PD-L1 antibody. As depicted in **Figure 7B**, anti-PD-L1 antibody successfully controlled tumor growth in the mice. Notably, h3D5-hIgG1 significantly inhibited MC38-hPD-L1 tumor growth. No significant changes in body weight or signs of toxicity were observed during the course of the study (**Figure 7C**). The group treated with h3D5-hIgG1 exhibited a significant survival advantage (**Figure 7D**).

## Discussion

After decades of effort, scientists have resolved the structure of a protein that only covers 17% of the amino acids in the human protein sequence (2). In their paper published in Nature, researchers demonstrated that AlphaFold2 could predict the structural locations of 58% of the amino acids in the human proteome with confidence, and the structure of 35.7% of amino acids was predicted with very high confidence. At the protein level, AlphaFold2 provided a reliable prediction of at least three-quarters of the amino acid sequences of 43.8% of the proteins. This large-scale accurate structure prediction by AlphaFold2 greatly expands the availability of rational drug design, which is primarily based on the discovery of protein-structured drugs (29).

Based on AlphaFold2’s predictions (30), the anti-PD-L1 antibody was successfully humanized in this study.

During the process of humanizing antibodies, when dealing with each parental nonhuman antibody framework, select a human germline framework sequence that fully retains all upper hydrophobic core residues, as a platform of residues directly underneath the CDRs. In cases where it is not feasible to identify a suitable human germline framework sequence meeting this criterion, it becomes necessary to perform back mutation on the upper hydrophobic core residues. Additionally, proceed to analyze the potential impact of other amino acid residues within the framework on the CDRs. Whenever possible, aim to undertake humanization modifications on these amino acid residues to align them with human germline framework sequences.

AlphaFold2 predicted the three-dimensional VH structure of the parental nonhuman antibody 3D5, which revealed that Lys66 interacted with Val65 and Phe63 in the HCDR2. In the human germline (31), the 66th amino acid residue is Arg. Mutating Lys66 in the parental nonhuman antibody 3D5 to Arg66 did not affect the CDR region’s interaction with Val65 and Phe63. Of the upper hydrophobic core residues, namely Vel48, Ala67, Leu69, Ala71, Ala78, and Gly94, they are subjected to back mutation during the humanization process. Alterations to them can impact the activity of antibodies.

AlphaFold2 also predicted the three-dimensional structure of VL of the parental nonhuman antibody 3D5. In the human germline, position 3 is Gln, and position 63 is Ser. The interaction between Val3, Gln3, and Ser26 in LCDR1 is similar in the three-dimensional structure. However, the interaction between Ser66 and Arg54 resulted in one less hydrogen bond than the interaction between Thr66 and Arg54 in the parental nonhuman antibody framework. Thr66 was still modified to Ser66, considering reducing immunogenicity. The final results showed that the modification of the amino acid residue did not affect the antibody’s binding activity.

To further observe whether the humanized antibody affects the binding of antigens, the 3D5/PD-L1 complex was modeled by AlphaFold2. The modified antibody h3D5 showed an expanded area of HCDR2 binding epitopes and interaction force with the antigen. 3D5 and h3D5 mainly bound to Epitope 2 and Epitope 3 of the PD-L1 protein. After modification, the binding epitope residues on the PD-L1 protein were only slightly changed. The results showed that the modified antibodies might have better binding ability and blocking ability due to the enhancement of antibody binding ability.

While AlphaFold2 demonstrates high accuracy in predicting individual proteins and antibodies. However, in our attempts to predict other antigen-antibody complexes using AlphaFold2, it has come to our attention that not all antigen-antibody complexes can be successfully predicted. As widely recognized, the self-learning capabilities of artificial intelligence heavily rely on a sufficiently robust database. The success in predicting PD-L1 antibody-antigen complexes might be attributed to the availability of existing antibody-antigen complex crystal structures within the PDB database, which could serve as valuable learning resources for AlphaFold2, thus enabling it to make predictions for this specific class of antibody-antigen complexes. It is important to acknowledge that artificial intelligence still has a significant journey ahead. Presently, predictions of antibody-antigen complexes should be regarded only as references, given the challenges and limitations encountered in the predictive process.

Nevertheless, the binding epitope predicted by AlphaFold2 exhibits similarity to the PD-L1 epitope that binds with PD-1. Furthermore, wet lab experiments have confirmed that 3D5 is capable of disrupting the interaction between PD-1 and PD-L1. We can utilize the AlphaFold2 predicted results as a reference and subsequently perform docking of the antibody antigen using shape complementarity and electrostatics. ZDOCK 3.0, has a scoring function that includes shape complementarity, electrostatics, and a pairwise atomic statistical potential developed using contact propensities of transient protein complexes. Based on the structure predicted by AlphaFold2, we performed docking of the antigen-antibody complex using ZDOCK (24). Using PRODIGY to predict the antibody affinity (26, 27), we found similar conclusions, that the humanized antibody h3D5 has better binding ability. Similarly, this conclusion should also be considered solely as a reference. AlphaFold2 has been of immense assistance in predicting the structure of antibodies, greatly facilitating the process of humanization.

Therapeutic antibodies that inhibit immune checkpoints are very effective means to enhance T cell responses, and they have been successfully exploited to treat patients with different types of cancers (16, 32). The h3D5-hIgG1 bound to human and cynomolgus monkey PD-L1 with high affinity and blocked the interaction of PD-L1 with PD-1. Furthermore, h3D5-hIgG1 also showed antitumor activity in syngeneic tumor models without additional modification.

In summary, with the help of AlphaFold2, the antibody could be rapidly and correctly mutated and modified, and an excellent PD-L1 antibody was successfully obtained. Compared to traditional humanization processes for anti-PD-L1 antibodies, our use of AlphaFold2 for antibody structure prediction offers a faster and more precise approach. This study provides a perspective on successful drug development assisted by artificial intelligence, which will become a trend in the future.

## Materials and methods

### Generation of mouse anti-PD-L1 antibody

Balb/C mice were immunized with PD-L1 Fc protein (Internal, 50μg/mouse) after the antigen was diluted to 1mg/mL and mixed with CFA or IFA (initial immunization with CFA and subsequent immunization with IFA). Protein immunization was repeated for more than 3 times at an interval of 2 weeks. After 14 days of the last immunization, PD-L1 Fc protein was injected intraperitoneally. After 3 days, spleen cells of mouse were taken for cell fusion and mouse spleen cells were fused with SP2/0 cells (ATCC) were electrofused in a 2:1 ratio and cultured in HAT(GIBCO, Code:H0262) medium in 96-well culture plates. After 10 days, hybridoma cell supernatant antibodies were screened.

### Antibody humanization

Align each parental nonhuman antibody framework sequence (heavy chain framework 1, 2, 3, and 4 and light chain framework 1, 2, 3, and 4) with the human germline framework sequences obtained through the protein database, such as IMGT (33). Choose an appropriate and closely related germline. Check the residues comprising the upper hydrophobic core within the immunoglobulin domain: these are located at positions 2, 4, 24, 27, and 29 in framework 1; 69, 71, 78, and 94 in framework 3 of the heavy chain, and at positions 2 and 4 in framework 1; and 64, 66, and 71 in framework 3 of the light chain of the parental nonhuman antibody framework sequence (residue positions are described in Kabat numbering).The antibody structure was predicted by AlphaFold2, and the interaction force was judged according to the distance between amino acid residues to carry out appropriate modification by pymol. Furthermore, AlphaFold2 was used to further predict the structure of the antibody antigen complex.

### ZDOCK

Input structures and options. On the initial submission page, users provide two input structures to be docked (one structure in the case of M-ZDOCK), either by uploading their own PDB files or by specifying PDB codes followed by selection of chains or biological assembly via dynamically generated checkboxes. Options include selection of ZDOCK version (3.0.2 or 2.3.2).Selection of blocking/contacting residues. The next step is selection of blocking (ZDOCK and M-ZDOCK) and contacting (ZDOCK only) residues for each submitted protein, which is aided by JMol visualization of each molecule that highlights selected residues for the user. Viewing results. Users are emailed a link to the results page on job completion, where randomly generated codes are used for results page names (to ensure privacy of users’ data). In addition to the ZDOCK output and pre-processed input PDB files, the results page features a JMol visualization of the top docking models and the center-of-mass positions of the top 500 ligands (the latter for ZDOCK only), as well as the capability to visualize (in JMol) or download any individual complex from the output. In addition to the top 10 models that are available as a user download, sets of predicted complexes can be generated using a Java program, or alternatively an executable file that is included with the download of the appropriate program (ZDOCK or M-ZDOCK).

### BLI assay(ForteBio Octet)

Chose appropriate sensor chip; Antibodies were diluted with running buffer(1× PBS+ 0.02% Tween20 + 0.1%BSA) to 5μg/mL and then captured onto a chip. Seven concentrations (200nM, 100nM50nM, 25nM, 12.5nM, 6.25nM) of human PD-L1 antigen. Glycine (10 mM, pH 1.5) used as regeneration buffer was injected following each dissociation phase.

### FACS assay

Human PD-L1-expressing 293T cells(Internal) were incubated with serial dilutions of test anti-PD-L1 antibodies at 4°C for 1 h. Testing antibodies were serially diluted (1:2) in wash buffer (1× PBS/1% BSA) starting from 20μg/ml. A PE-labeled goat anti-human IgG was used to detect the binding of anti-PD-L1 antibodies to the cells The mean fluorescence intensity of cells was measured by a flow cytometer and analyzed by FlowJo.

### Reporter assays

In the report cell line analysis experiment using two-cell co-culture system including the signal sensor cell, jurkat, that expresses the chimeric PD-1 receptor and NFAT-luciferase, and the signal sending cell, 293T/PD-L1/OKT3. The Reporter assay was set up in 96-well round-bottom plates using complete RPMI-1640 medium. Jurkat/PD-1 cells, various concentrations of antibodies, and 293T/PD-L1/OKT3 cells were added to the plates at appropriate ratios. The plates were incubated at 37°C in 5% CO2. Luciferase was detected after 6 hours.

### Mixed lymphocyte reaction assay

Isolated PBMCs were cultured in complete RPMI-1640 medium (containing 10% FBS) supplemented with 100 U recombinant human IL-2 (34). CD4+ T cells were isolated from human PBMCs. Purified CD4+ T cells were cocultured with immature or mature allogeneic DCs. The MLR assay was set up in 96-well round-bottom plates using complete RPMI- 1640 medium. CD4+ T cells, various concentrations of antibodies, and allogeneic DCs were added to the plates at appropriate ratios. The plates were incubated at 37°C in 5% CO2. IL-2 and IFN-γ levels were determined with ELISA assay on day 5 (35).

### *In vivo* efficacy study in tumor models

The animal model of MC38-PD-L1 cells was established by hPD-L1 knock-in mice (8 weeks, female) and the efficacy of anti-PD-L1 antibody was verified *in vivo*. MC38-PD-L1 cells (2.5×10^6^ cells/mL) were suspended by PBS and inoculated subcutaneously on the right side of hPD-L1 knock-in mice. When the mean tumor volume reached about 100 mm^3^, the groups were divided and intraperitoneally injected with 10 mg/kg anti-PD-L1 antibody or equal volume of solvent (PBS) control, twice a week. The mice were fed normally after administration, and the survival status of the mice was observed, and the body weight and tumor volume of the mice were recorded.

## Data availability statement

The original contributions presented in the study are included in the article/supplementary material. Further inquiries can be directed to the corresponding author.

## Ethics statement

Ethical approval was not required for the studies on humans in accordance with the local legislation and institutional requirements because only commercially available established cell lines were used. The animal study was approved by Tianjin University Animal Ethics Committee, Tianjin University. The study was conducted in accordance with the local legislation and institutional requirements.

## Author contributions

KD: Conceptualization, Investigation, Software, Writing – original draft, Writing – review & editing. HH: Formal Analysis, Funding acquisition, Project administration, Resources, Supervision, Writing – review & editing.

## References

[1] Jumper J Evans R Pritzel A Green T Figurnov M Ronneberger O. Highly accurate protein structure prediction with AlphaFold. Nature (2021) 596(7873):583–9. doi: 10.1038/s41586-021-03819-2 PMC8371605 34265844

[2] Tunyasuvunakool K Adler J Wu Z Green T Zielinski M Žídek A. Highly accurate protein structure prediction for the human proteome. Nature (2021) 596(7873):590–6. doi: 10.1038/s41586-021-03828-1 PMC8387240 34293799

[3] Kryshtafovych A Schwede T Topf M Fidelis K Moult J. Critical assessment of methods of protein structure prediction (CASP)—Round XIII. Proteins: Structure Function Bioinf (2019) 87(12):1011–20. doi: 10.1002/prot.25823 PMC6927249 31589781

[4] Pereira J Simpkin AJ Hartmann MD Rigden DJ Keegan RM Lupas AN. High-accuracy protein structure prediction in CASP14. Proteins: Structure Function Bioinf (2021) 89(12):1687–99. doi: 10.1002/prot.26171 34218458

[5] Yang J Anishchenko I Park H Peng Z Ovchinnikov S Baker D. Improved protein structure prediction using predicted interresidue orientations. Proc Natl Acad Sci USA (2020) 117:1496–503. doi: 10.1073/pnas.1914677117 PMC6983395 31896580

[6] Greener JG Kandathil SM Jones DT. Deep learning extends *de novo* protein modelling coverage of genomes using iteratively predicted structural constraints. Nat Commun (2019) 10:3977. doi: 10.1038/s41467-019-11994-0 31484923 PMC6726615

[7] Michel M Menéndez Hurtado D Uziela K Elofsson A. Large-scale structure prediction by improved contact predictions and model quality assessment. Bioinformatics (2017) 33:i23–9. doi: 10.1093/bioinformatics/btx239 PMC5870574 28881974

[8] Ovchinnikov S Kinch L Park H Liao Y Pei J Kim DE. Large-scale determination of previously unsolved protein structures using evolutionary information. eLife (2015) 4:e09248. doi: 10.7554/eLife.09248 26335199 PMC4602095

[9] Zhang J Yang J Jang R Zhang Y. GPCR-I-TASSER: a hybrid approach to G protein-coupled receptor structure modeling and the application to the human genome. Structure (2015) 23:1538–49. doi: 10.1016/j.str.2015.06.007 PMC4526412 26190572

[10] Bender BJ Marlow B Meiler J. Improving homology modeling from low-sequence identity templates in Rosetta: a case study in GPCRs. PloS Comput Biol (2020) 16:e1007597. doi: 10.1371/journal.pcbi.1007597 33112852 PMC7652349

[11] Drew K Winters P Butterfoss GL Berstis V Uplinger K Armstrong J. The Proteome Folding Project: proteome-scale prediction of structure and function. Genome Res (2011) 21:1981–94. doi: 10.1101/gr.121475.111 PMC3205581 21824995

[12] Xu D Zhang Y. Ab initio structure prediction for Escherichia coli: towards genome-wide protein structure modeling and fold assignment. Sci Rep (2013) 3:1895. doi: 10.1038/srep01895 23719418 PMC3667494

[13] Waterhouse A Bertoni M Bienert S Studer G Tauriello G Gumienny R. SWISS-MODEL: homology modelling of protein structures and complexes. Nucleic Acids Res (2018) 46:W296–303. doi: 10.1093/nar/gky427 PMC6030848 29788355

[14] Mullard A. New checkpoint inhibitors ride the immunotherapy tsunami. Nat Rev Drug Discovery (2013) 12:489–92. doi: 10.1038/nrd4066 23812256

[15] Mellman I Coukos G Dranoff G. Cancer immunotherapy comes of age. Nature (2011) 480:480–9. doi: 10.1038/nature10673 PMC3967235 22193102

[16] Pardoll DM. The blockade of immune checkpoints in cancer immunotherapy. Nat Rev Cancer (2012) 12:252–64. doi: 10.1038/nrc3239 PMC4856023 22437870

[17] Okazaki T Honjo T. The PD-1-PD-L pathway in immunological tolerance. Trends Immunol (2006) 27:195–201. doi: 10.1016/j.it.2006.02.001 16500147

[18] Carreno BM Collins M. The B7 family of ligands and its receptors: new pathways for costimulation and inhibition of immune responses. Annu Rev Immunol (2002) 20:29–53. doi: 10.1146/annurev.immunol.20.091101.091806 11861596

[19] Shi L Chen S Yang L Li Y. The role of PD-1 and PD-L1 in T-cell immune suppression in patients with hematological Malignancies. J Hematol Oncol (2013) 6:1–6. doi: 10.1186/1756-8722-6-74 24283718 PMC3851976

[20] Iwai Y Ishida M Tanaka Y Okazaki T Honjo T Minato N. Involvement of PD-L1 on tumor cells in the escape from host immune system and tumor immunotherapy by PD-L1 blockade. Proc Natl Acad Sci USA (2002) 99:12293–7. doi: 10.1073/pnas.192461099 PMC129438 12218188

[21] Wherry EJ. T cell exhaustion. Nat Immunol (2011) 12:492–9. doi: 10.1038/ni.2035 21739672

[22] Sakuishi K Apetoh L Sullivan JM Blazar BR Kuchroo VK Anderson AC. Targeting Tim-3 and PD-1 pathways to reverse T cell exhaustion and restore antitumor immunity. J Exp Med (2010) 207:2187–94. doi: 10.1084/jem.20100643 PMC2947065 20819927

[23] Zang X Allison JP. The B7 family and cancer therapy: costimulation and coinhibition. Clin Cancer Res (2007) 13:5271–9. doi: 10.1158/1078-0432.CCR-07-1030 17875755

[24] Pierce BG Wiehe K Hwang H Kim BH Vreven T Weng Z. ZDOCK server: interactive docking prediction of protein-protein complexes and symmetric multimers. Bioinformatics (2014) 30(12):1771–3. doi: 10.1093/bioinformatics/btu097 PMC4058926 24532726

[25] Mintseris J Pierce B Wiehe K Anderson R Chen R Weng Z. Integrating statistical pair potentials into protein complex prediction. Proteins: Structure Function Bioinf (2007) 69(3):511–20. doi: 10.1002/prot.21502 17623839

[26] Vangone A Bonvin AMJJ. Contact-based prediction of binding affinity in protein-protein complexes. eLife (2015) 4:e07454. doi: 10.7554/eLife.07454 26193119 PMC4523921

[27] Xue L Rodrigues J Kastritis P A.M.J.J.* B Vangone A. PRODIGY: a web-server for predicting the binding affinity in protein-protein complexes. Bioinformatics (2016) 32(23):3676–8. doi: 10.1093/bioinformatics/btw514 27503228

[28] Brown JA Dorfman DM Ma FR Sullivan EL Munoz O Wood CR. Blockade of programmed death-1 ligands on dendritic cells enhances T cell activation and cytokine production. J Immunol (2003) 170:1257–66. doi: 10.4049/jimmunol.170.3.1257 12538684

[29] Borkakoti N Thornton JM. AlphaFold2 protein structure prediction: Implications for drug discovery. Curr Opin Struct Biol (2023) 78:102526. doi: 10.1016/j.sbi.2022.102526 36621153 PMC7614146

[30] Mirdita M Schütze K Moriwaki Y Heo L Ovchinnikov S Steinegger M. ColabFold: making protein folding accessible to all. Nat Methods (2022) 19(6):679–82. doi: 10.1038/s41592-022-01488-1 PMC9184281 35637307

[31] Hsieh YC Liao JM Chuang KH Ho KW Hong ST Liu HJ. A universal in silico V (D) J recombination strategy for developing humanized monoclonal antibodies. J Nanobiotechnol (2022) 20(1):1–11. doi: 10.1186/s12951-022-01259-2 PMC8805405 35101043

[32] Strome SE Dong H Tamura H Voss SG Flies DB Tamada K. B7-H1 blockade augments adoptive T-cell immunotherapy for squamous cell carcinoma. Cancer Res (2003) 63(19):6501–5. 14559843

[33] Kuramochi T Igawa T Tsunoda H Hattori K. Humanization and simultaneous optimization of monoclonal antibody. Hum Monoclonal Antibodies: Methods Protoc (2014), 123–37. doi: 10.1007/978-1-62703-586-6_7 24037839

[34] Li F Li J Yin K Zhang J Li ZH Lu L. CS1003, a novel human and mouse cross-reactive PD-1 monoclonal antibody for cancer therapy. Acta Pharmacologica Sin (2021) 42(1):142–8. doi: 10.1038/s41401-020-0422-6 PMC7921591 32467569

[35] Li Y Carpenito C Wang G Surguladze D Forest A Malabunga M. Discovery and preclinical characterization of the antagonist anti-PD-L1 monoclonal antibody LY3300054. J Immunother Cancer (2018) 6(1):1–14. doi: 10.1186/s40425-018-0329-7 29712568 PMC5925824

